# Optical coherence tomography biomarkers in patients with macular edema secondary to retinal vein occlusion treated with dexamethasone implant

**DOI:** 10.1186/s12886-022-02415-w

**Published:** 2022-04-26

**Authors:** Verónica Castro-Navarro, Clara Monferrer-Adsuara, Catalina Navarro-Palop, Javier Montero-Hernández, Enrique Cervera-Taulet

**Affiliations:** Ophthalmology Department, Consorci Hospital General Universitari de Valencia, Avenida Tres Cruces s/n, 46016 Valencia, Spain

**Keywords:** Macular edema, Central retinal vein occlusion, Branch retinal vein occlusion, Intravitreal dexamethasone implant, OCT

## Abstract

**Purpose:**

To evaluate the impact of optical coherence tomography (OCT) biomarkers on intravitreal dexamethasone (DEX) implant clinical outcomes in patients with macular edema secondary to retinal vein occlusion (RVO-ME).

**Methods:**

Retrospective study conducted on a cohort of patients with RVO-ME, either naïve or previously treated, who underwent treatment with DEX implant and had a follow-up of 6 months. Anatomic success was defined as a central retinal thickness (CRT) < 250 μm or a relative reduction of CRT ≥10% from baseline. The primary endpoint was the mean change in CRT from baseline to month-6. Secondary end-points included changes in BCVA, the impact of baseline OCT biomarkers on functional and anatomic outcomes; and the impact of treatment on the different OCT biomarkers. OCT biomarkers associated with functional and anatomic outcomes were estimated using a logistic regression model.

**Results:**

Fifty-seven eyes were included in the study. Baseline CRT was significantly decreased from 567.6 ± 226.2 μm to 326.9 ± 141.0 μm at month-6 (*p* < 0.0001). Baseline BCVA was significantly lower in the eyes with disrupted external limiting membrane (ELM) (mean 40.3 ± 21.3 letters) than in those with non-disrupted (mean 68.6 ± 10.7 letters) or partially-disrupted ELM (mean 59.6 ± 13.2 letters), *p* = 0.0001 and *p* = 0.0011, respectively. Baseline BCVA was significantly lower in eyes with > 20 hyperreflective foci (HRF) than in those with < 10 HRF (*p* = 0.0388). The eyes with disorganization of the retinal inner layers (DRIL) had lower baseline BCVA than those without DRIL (Hodges-Lehmann median difference: − 12.0 letters, 95% CI: − 25.0 to − 5.0 letters, *p* = 0.0042). At month-6, 26 (45.6%); 24 (42.1%), and 20 (35.1%) eyes achieved a BCVA improvement ≥5, ≥10, and ≥ 15 letters respectively. Forty (70.2%) eyes were classified as anatomic success at month-6. Logistic regression analysis found none factor significantly associated with success in the multivariate analysis.

**Conclusions:**

The results of this study suggested a positive impact of DEX on CRT and BCVA in eyes with RVO-ME. No OCT-biomarkers were identified as predictors of clinical-outcomes. Additionally, presence of DRIL, presence of HRF (> 20), or disrupted ELM were significantly associated with worse baseline BCVA.

## Introduction

Retinal vein occlusion (RVO) is a prevalent and disabling condition that may affect approximately 0.5% of people aging from 31 to 101 years [1]. RVO can be divided into two main types: branch retinal vein occlusion (BRVO) and central retina vein occlusion (CRVO) according to anatomic location of occlusion. BRVO is more common and has a better prognosis than CRVO [1, 2].

The introduction of intravitreal therapies has entailed a significant improvement in functional and anatomic outcomes among patients with macular edema secondary to RVO (RVO-ME) [3].

Corticosteroids, beside inhibiting the vascular endothelial growth factor (VEGF) pathway [4, 5], are able to downregulate other proinflammatory mediators [6].

Sustained release intravitreal dexamethasone (DEX) implant (Ozurdex; Allergan, Inc., Irvine, CA, USA) was introduced as a therapeutic option for treating RVO-ME patients. The results of the GENEVA study found significantly better functional and anatomic outcomes after DEX administration in eyes with RVO-ME [7, 8].

Optical coherence tomography (OCT) has dramatically changed the approach of retinal diseases. It allows quantitative and qualitative analysis of imaging biomarkers such as intraretinal hyperreflective foci (HRF); disorganization of the retinal inner layers (DRIL); external limiting membrane (ELM) disruption; ellipsoid zone (EZ) disruption; and retinal and choroidal thickness [9–14].

We have evidence suggesting that OCT biomarkers may help to predict treatment clinical outcomes. However, predictive value of OCT biomarkers varies among studies, likely because of differences in protocols, study populations, and treatment patterns. This issue may finally result in controversy regarding the validity of these findings [9–16].

Identification of imaging features on OCT that may predict clinical outcomes, is crucial for determining the best therapeutic approach.

In an attempt to identify the associations between OCT biomarkers and treatment outcomes, the current study evaluated quantitative and qualitative OCT features in a cohort of patients, either treatment naïve or previously treated, with RVO-ME who underwent treatment with DEX implant. Additionally, we also evaluated the effectiveness of DEX implant on these features.

## Methods

This was a retrospective study conducted on a cohort of patients with RVO, either BRVO or CRVO, who received treatment with DEX implant between January 2014 and September 2018.

The study complied with the tenets of the Declaration of Helsinki and was approved by the Ethics Committee of the Hospital General de Valencia (Protocol number: HGV-DEX-2020-01; Register 180/2020), which waived the need for informed consent for participating in the study.

### Patients

Eligible patients had a clinical diagnosed of ME secondary to RVO, defined as a central retinal thickness (CRT) ≥ 250 μm measured with OCT, either naïve or previously treated, who underwent treatment with DEX implant and had a minimum follow-up of 6 months.

Patients with ME secondary to other any condition different to RVO (i.e., diabetes, uveitis, etc.), any intercurrent disease, either ophthalmic or systemic, that could prevent visual acuity (VA) recovery, or uncontrolled glaucoma (defined as a medically treated intraocular pressure > 24 mmHg) were excluded of the study.

Best corrected Visual acuity (BCVA) was measured using the ETDRS scale chart located at 4 m of the patient.

### OCT measurements

All the patients underwent OCT imaging using the 3D-TopCon (3D OCT-2000 Spectral Domain OCT, Topcon Medical Systems, Inc., Oakland, USA). A three-dimensional scan protocols composed of 512 A-scans for each B-scans was used for macular measurements. Macular scans were performed with horizontal scanning protocols covering a 9 × 9 mm area centered on the fovea. Foveal thickness was defined as the average thickness in the central 1000-μm diameter of the Early Treatment Diabetic Retinopathy Study layout.

OCT images were evaluated by a trained ophthalmologist (CMA-retina specialist), who was blind to patient information (both demographic and clinic).

RVO-ME was classified, according to the Otani et al. classification [17], in sponge-like retinal swelling (SLRS); cystoid ME (CME); and serous retinal detachment (SRD). If SLRS was combined with CME or SRD, the pattern was classified as either CME or SRD, as appropriate, and when SLRS, CME, and SRD were all present, the type was classified as SRD. Other biomarkers assessed on Spectral Domain OCT (SD-OCT) were: the presence of subretinal fluid (SRF) and its height, cystoid changes in the outer nuclear layer (ONL) and in the inner nuclear layer (INL) and maximal cyst size; presence of septae; presence of DRIL, presence of HRF (number and location); subfoveal choroidal thickness (CST), and choroidal thickness measured at 500 μ and 1500 μ nasal (CTN) and temporally (CTT); and ELM continuity. The classification of the ELM disruption area is based on texture and morphologic features. Three different scenarios were arbitrarily considered, namely (1) Non-disrupted, whether discontinuity was less than 500 μm; (2) Partially disrupted, if it was between 500 μm and 1000 μm; and (3) disrupted when discontinuity was greater than 1000 μm.

The number of HRF was arbitrarily defined as Few (≥ 2 to ≤10); Moderate (> 10 to ≤20); or Many (> 20); and its locations as between the internal limiting membrane and the INL; between the outer plexiform layer and ELM; and in all retinal layers.

#### Outcomes

##### Definitions

Treatment naïve patient was defined as a patient who, up to that moment, had never received any treatment (pharmacological, laser, and/or surgical).

Anatomic success was defined as a CRT < 250 or a relative reduction of CRT ≥ 10% from baseline [14].

Patients received anti-VEGF as rescue therapy, unless there were contraindications, including heart problems in the last 6 months, bad or poor visit regimen compliance, or a previously failed anti-VEGF regime, in such cases patients received an additional DEX implant. Rescue therapy was administered on a pro re nata regime, according to specific retreatment criteria, including loss of VA of more than 5 ETDRS letters, increase in CRT of > 50 μm, and/or presence of intraretinal (IRF)/subretinal (SRF) fluid compared to the previous visit [18].

#### Outcomes

The primary endpoint was the mean change in CRT from baseline to month-6.

Secondary outcomes included mean change in BCVA; proportion of eyes gaining ≥5, ≥ 10, and ≥ 15 letters in BCVA; the impact of the different OCT biomarkers on functional and anatomic outcomes; and the impact of treatment on the different OCT biomarkers.

### Statistical analysis

MedCalc® Statistical Software version 19.8 (MedCalc Software Ltd., Ostend, Belgium; https://www.medcalc.org; 2021) and the SPSS IBM Corp. Released 2019 (IBM SPSS Statistics for Windows, Version 26.0. Armonk, NY: IBM Corp) were used to perform the statistical analysis.

Although sample size was not calculated before the study, we have conducted a post hoc analysis for testing the adequacy of sample. The post hoc power analyses were determined for an alpha level of 0.05, the study sample size, and the effect size observed in the study [19].

Descriptive statistics number (percentage); mean and (SD); mean and 95% confidence interval (95% CI); median and interquartile range (IqR), or median (95% CI) were used, as appropriate.

Of the total amount of follow-up measurements, 4% was missing and they were allocated using an algorithm of multiple imputation [20].

Data were tested for normal distribution using a D’Agostino-Pearson test.

A repeated measures ANOVA or a Friedman’s two-way analysis test as appropriate, were used to assess changes in BCVA and CRT within the groups throughout the study.

The one-way ANOVA test or the Kruskal-Wallis test were used to compare differences between groups. Post hoc analysis for pair wise comparisons were done with the Scheffé’s method (ANOVA) or the Conover method (Kruskal-Wallis).

The Mann–Whitney U test was used in the evaluation of the baseline clinical and demographic parameters between naïve and previously treated eyes and between CRVO and BRVO eyes.

A logistic regression model, for both univariate and multivariate analysis, was used to estimate and test factors for their association with success criterion. Factors associated with success in the univariate analysis at *p* ≤ 0.1 were included in the multivariate analysis. A backward strategy was adopted, with a statistically significant cut-off for variable screening of 0.05.

The analysis of covariance (ANCOVA) was used in the evaluation of the changes in BCVA and CRT between the different diagnosis and ELM status. The first model included “diagnosis” as a factor and age, sex, duration of RVO-ME, treatment status (naïve or previously treated), and ELM status as covariates. The second model included “Treatment status” as factor and age, sex, duration of macular edema, diagnosis (CRVO or BRVO), type of macular edema (ME), and ELM status as covariates. The third model included “ELM status” (classified as non-disrupted, partially disrupted, and disrupted) as factor and age, sex, duration of RVO-ME, treatment status, and diagnosis as covariates.

For evaluating the differences between groups in the ANCOVA analysis, Bonferroni corrected *P*-value was provided.

Categorical variables were compared using a Chi-square test and a Fisher’s exact test, as needed. *P* value of less than 0.05 was considered significant.

## Results

### Baseline demographic and clinical characteristics

Fifty-seven eyes (29 treatment naïve and 28 previously treated) fulfilled the inclusion/exclusion criteria requirements. Fifteen (26.3%) eyes were diagnosed with CRVO and 42 (73.7%) ones with BRVO. In the overall study sample, mean age was 72.4 ± 9.3 years.

With the exception of the number of intravitreal injections previously administered (when comparing treatment naïve versus [vs] previously treated, *p* < 0.0001) there were no significant differences in any of the baseline study variables.

Median (IqR) duration of RVO-ME before treatment was 32.0 (8.0 to 83.0) days.

The Table 1 summarizes the main baseline clinical and demographic characteristics of the study population.

**Table 1:** Baseline demographic and clinical characteristics in the overall study population; in the central retinal vein occlusion (CRVO) and branch retinal vein occlusion (BRVO) samples; and in the treatment naïve and previously treated patients

Variable	Overall (***n*** = 57)	CRVO (***n*** = 15)	BRVO (***n*** = 42)	P^*^	Naïve (***n*** = 31)	Previously treated (***n*** = 26)	P^*^
Type of RVO, n (%)							
CRVO	15 (26.3)	N.A.	N.A.	N.A.	9 (21.0)	6 (23.1)	0.7647
BRVO	42 (73.7)				22 (71.0)	20 (76.9)	
Treatment status, n (%)							
Naïve	31 (54.4)	9 (60.0)	22 (52.4)	0.7647^a^	N.A.	N.A.	N.A.
Previously treated	26 (45.6)	6 (40.0)	20 (47.6)				
Age, years							
Mean (SD)	72.4 (9.3)	70.6 (9.1)	73.0 (9.4)	0.3994	72.3 (10.5)	72.5 (7.9)	0.8225
95% CI	69.9 to 74.9	65.6 to 75.7	70.1 to 76.0		68.4 to 76.1	69.3 to 75.7	
Sex, n (%)							
Women	34 (59.6)	10 (66.7)	24 (57.1)	0.5224	19 (61.3)	15 (57.7)	0.7846
Men	23 (40.4)	5 (33.3)	18 (42.9)		12 (38.7)	11 (42.3)	
Eye, n (%)							
Right	25 (43.9)	5 (33.3	20 (47.6)	0.3428	14 (45.2)	11 (42.3)	0.8303
Left	32 (56.1)	10 (66.7)	22 (52.4)		17 (54.8)	15 (57.7)	
NOII							
Mean (SD)	1.3 (1.7)	1.1 (1.5)	1.4 (1.8)	0.5850	0.0	2.9 (1.4)	< 0.0001
95% CI	0.8 to 1.8	0.2 to 1.9	0.8 to 1.9		0.0 to 0.0	2.3 to 3.4	
Type of RVO-ME, n (%)							
SLRS	2 (3.5)	1 (6.7)	1 (2.4)	0.3056^b^	2 (6.5)	0 (0.0)	0.1474^b^
CME	20 (35.1)	3 (20.0)	17 (40.5)		6 (19.4)	14 (53.8)	
SRD	35 (61.4)	11 (73.3)	24 (57.1)		23 (74.2)	12 (46.2)	
Duration of RVO-ME, days							
Mean (SD)	61.3 (81.6)	60.7 (70.8)	61.5 (85.9)	0.8632	62.5 (94.3)	59.9 (65.2)	0.5424
95% CI	39.7 to 83.0	21.5 to 99.9	34.8 to 84.3		27.9 to 97.1	33.6 to 86.2	
BCVA^**^							
Mean (SD)	55.2 (19.4)	55.5 (15.7)	55.1 (20.7)	0.6738	55.0 (19.0)	55.5 (20.3)	0.7524
95% CI	50.1 to 60.4	46.8 to 64.2	48.7 to 61.6		48.1 to 62.0	47.3 to 63.7	
CRT, μm							
Mean (SD)	567.6 (226.2)	549.8 (158.7)	573.9 (247.3)	0.8420	617.7 (280.0)	507.9 (117.1)	0.2846
95% CI	507.6 to627.6	461.9 to 637.7	496.9 to 651.0		515.0 to 720.4	460.6 to 555.1	
NMCST500, μm							
Mean (SD)	175.1 (53.6)	180.2 (53.0)	173.7 (54.3)	0.6570	180.2 (54.6)	169.1 (52.8)	0.3610
95% CI	160.9 to 189.4	150.9 to 209.5	156.4 to 190.3		160.2 to 200.2	147.8 to 190.4	
NMCST1500, μm							
Mean (SD)	180.6 (58.4)	188.3 (60.6)	177.9 (78.1)	0.5742	183.5 (58.9)	177.2 (58.7)	0.5859
95% CI	165.1 to 196.1	154.7 to 221.8	159.8 to 196.0		161.9 to 205.1	153.5 to 200.9	
TMCST500, μm							
Mean (SD)	189.6 (76.5)	205.5 (118.5)	183.9 (55.4)	0.9278	186.4 (65.5)	193.5 (89.1)	0.6137
95% CI	169.3 to 209.9	139.8 to 275.1	166.7 to 201.2		162.3 to 210.4	157.5 to 229.4	
TMCST1500, μm							
Mean (SD)	184.1 (56.5)	194.1 (75.7)	180.5 (48.4)	0.5804	189.2 (61.4)	178.0 (50.4)	0.2866
95% CI	169.1 to 199.1	152.1 to 236.0	165.5 to 195.6		166.7 to 211.8	157.6 to 198.4	
CST, μm							
Mean (SD)	184.1 (57.0)	184.1 (67.3)	184.1 (53.7)	0.9494	186.8 (54.6)	180.8 (60.6)	0.5749
95% CI	169.0 to 199.2	146.9 to 221.4	167.3 to 200.8		166.8 to 206.9	156.3 to 205.3	
HRF, n (%)							
< 10	16 (28.1)	3 (20.0)	13 (31.0)	0.3247^b^	8 (25.8)	8 (30.8)	0.5098^b^
10–20	23 (40.4)	11 (73.3)	12 (28.6)		12 (38.7)	11 (42.3)	
> 20	18 (31.6)	1 (6.7)	17 (40.5)		11 (35.5)	7 (26.9)	
DRIL, n (%)							
Yes	34 (59.6)	10 (66.7)	24 (57.1)	0.5582^a^	20 (64.5)	14 (53.8)	0.4323^a^
No	23 (40.4)	5 (33.3)	18 (42.9)		11 (35.5)	12 (46.2)	
Cysts volume, μm^3^							
Mean (SD)	317.9 (145.4)	285.7 (121.8)	329.7 (152.8)	0.3268	318.9 (154.6)	316.9 (137.1)	0.9084
95% CI	279.0 to 356.9	218.2 to 353.1	281.5 to 378.0		261.2 to 376.6	261.5 to 372.2	
Cysts, n (%)							
< 100 μm	2 (3.6)	1 (6.7)	1 (2.4)	0.2838^b^	1 (3.3)	1 (3.8)	0.4658^b^
100–200 μm	12 (21.4)	5 (33.3)	7 (17.1)		8 (26.7)	4 (15.4)	
> 200 μm	42 (75.0)	9 (60.0)	33 (80.5)		21 (70.0)	21 (80.8)	
ELM, n (%)							
Non-disrupted	13 (22.8)	2 (13.3)	11 (26.2)	0.2059^b^	5 (16.1)	8 (30.8)	0.6727^b^
Partially disrupted	24 (42.1)	6 (40.0)	18 (42.9)		16 (51.6)	8 (30.8)	
Disrupted	20 (35.1)	7 (46.7)	13 (31.0)		10 (32.39	10 (38.5)	
SRF, n (%)							
Yes	35 (61.4)	11 (73.3)	24 (57.1)	0.3600^a^	23 (74.2)	12 (46.2)	0.0548^a^
No	22 (38.6)	4 (26.7)	18 (42.9)		8 (25.8)	14 (53.8)	
SRF, μm							
Mean (SD)	147.1 (112.2)	103.8 (38.8)	166.9 (129.0)	0.1097	158.4 (129.6)	125.3 (67.1)	0.3943
95% CI	108.5 to 185.6	77.8 to 129.9	112.4 to 221.4		102.3 to 214.4	82.7 to 168.0	

*RVO* Retinal vein occlusion, *CRVO* Central retinal vein occlusion, *BRVO* Branch retinal vein occlusion, *NA* Not applicable, *SD* Standard deviation, *95% CI* 95% Confidence interval, *NOII* Number of intravitreal injections, *RVO* retinal vein occlusion, *ME* macular edema, *SLRS* Sponge-like retinal swelling, *CME* Cystoid macular edema, *SRD* Serous retinal detachment, *CRT* Central retinal thickness, *NMCST* Nasal mean choroidal subfoveal thickness, *TMCST* Temporal mean choroidal subfoveal thickness, *500* ring of 500 μm, *1500* Ring of 1500 μm, *CST* Choroidal subfoveal thickness, *HRF* Hyperreflective foci, *DRIL* Disorganization of retinal inner layers, *ELM* External limiting membrane, *SRF* Serous retinal fluid

^a^Fisher exact test

^b^Chi-squared from trend test

^*^Mann-Whitney test

^**^Letters in the Early Treatment Diabetic Retinopathy Study (ETDRS) charts

### Baseline best corrected visual acuity

Baseline BCVA was significantly lower in the eyes with disrupted ELM (mean 40.3 ± 21.3 letters) than in those with non-disrupted (mean 68.6 ± 10.7 letters) or partially-disrupted ELM (mean 59.6 ± 13.2 letters), *p* = 0.0001 and *p* = 0.0011, respectively; and significantly greater in non-disrupted group than in the partially disrupted one (0.0370).

The presence of SRF was not associated with worse BCVA at baseline (Hodges-Lehmann median difference: 0.0; 95% CI: − 10.0 to 10.0, *p* = 0.9934).

The eyes with DRIL had a lower BCVA at baseline than those without DRIL (Hodges-Lehmann median difference: − 12.0 letters, 95% CI: − 25.0 to − 5.0 letters, *p* = 0.0042). Moreover, baseline BCVA was significantly lower in eyes with more than 20 HRF than in those with less than 10 HRF (*p* = 0.0388, Kruskal-Wallis test).

### Changes in BCVA

At month-6, 26 (45.6%); 24 (42.1%), and 20 (35.1%) eyes achieved a BCVA improvement ≥5, ≥10, and ≥ 15 letters respectively. However, 20 (35.1%) eyes experienced a BCVA worsening ≥5 letters. The Table 2 shows the proportion of eyes achieving certain change in BCVA according to different baseline characteristics.

**Table 2:** Change in best corrected visual acuity (BCVA) according to different baseline characteristics

	Change in BCVA from baseline to month 6^**c**^, n (%)				
	≥ 5	≥ 10	≥ 15	≤5	***p***^**a**^
Diagnosis					
CRVO	5 (19.2)	5 (20.8)	5 (25.0)	9 (42.9)	0.0728^b^
BRVO	21 (80.8)	19 (79.2)	15 (75.0)	12 (57.1)	
Treatment status					
Naïve	18 (69.2)	18 (75.0)	14 (70.0)	9 (42.9)	0.0690^b^
Previously treated	8 (30.8)	6 (25.0)	6 (30.0)	12 (57.1)	
ME duration^d^					
≤ 32 days	10 (38.5)	9 (37.5)	9 (45.0)	13 (61.9)	0.1010^b^
> 32 days	16 (61.5)	15 (62.5)	11 (55.0)	8 (38.1)	
Type of RVO-ME					
SLRS	0 (0.0)	0 (0.0)	0 (0.0)	2 (9.5)	0.1877
CME	8 (30.8)	7 (29.4)	8 (40.0)	9 (42.9)	
SRD	18 (69.2)	17 (70.8)	12 (60.0)	10 (47.6)	
HRF, n (%)					
< 10	5 (19.2)	4 (16.7)	5 (25.0)	6 (28.6)	0.7126
10–20	14 (53.8)	13 (54.2)	7 (35.0)	7 (33.3)	
> 20	7 (26.9)	7 (29.2)	8 (40.0)	8 (38.1)	
ELM, n (%)					
Non-disrupted	6 (23.1)	6 (25.0)	3 (15.0)	4 (19.0)	0.3960
Partially disrupted	15 (57.7)	13 (54.2)	10 (50.0)	7 (33.3)	
Disrupted	5 (19.2)	5 (20.8)	7 (35.0)	10 (47.6)	
DRIL, n (%)					
No	9 (34.6)	8 (33.3)	5 (25.0)	11 (52.4)	0.3391^b^
Yes	17 (65.4)	16 (66.7)	15 (75.0)	10 (47.6)	
Cysts, n (%)^e^					
< 100 μm	0 (0.0)	0 (0.0)	0 (0.0)	2 (10.0)	0.2014
100–200 μm	7 (26.9)	6 (25.0)	3 (15.0)	3 (15.0)	
> 200 μm	19 (73.1)	18 (75.0)	17 (5.5)	15 (75.0)	

*Abbreviations*: *BCVA* Best corrected visual acuity, *CRVO* Central retinal vein occlusion, *BRVO* Branch retinal vein occlusion, *EM* Macular edema, *RVO-ME* Macular edema secondary to retinal vein occlusion, *SLRS* Sponge-like retinal swelling, *CME* Cystoid macular edema, *SRD* Serous retinal detachment, *HRF* Hyperreflective foci, *ELM* External limiting membrane, *DRIL* Disorganization of the retinal inner layers

^a^Chi-squared test

^b^Chi-squared for trend test

^c^Letters in the Early Treatment Diabetic Retinopathy Study (ETDRS) charts

^d^Median split

^e^Fifty-six eyes

No differences were observed between any of the study variables either between CRVO and BRVO. Nevertheless, BCVA improvement was significantly greater in the treatment naïve eyes than in the previously treated ones (median difference 18.0; 95% CI: 4.0 to 35.0, *p* = 0.0129 (Table 3).

**Table 3:** Overview of the unadjusted mean changes from baseline to the last follow-up visit at month 6 between eyes with central retinal vein occlusion related macular edema (CVRO-ME) and those with macular edema secondary to branch retinal vein occlusion (BRVO-ME), and between treatment-naïve eyes and those previously treated

**Variables**	**Overall (*n*** **= 57)**					
	**Mean difference (95% CI) from baseline**					***P***^**b**^
BCVA	2.0 (−5.7 to 9.7)					0.6114
CRT, μm	− 240.7 (− 318.4 to − 162.9)					< 0.0001
CST, μm	1.7 (−17.8 to 21.3)					0.8594
NMCST500, μm	12.6 (−5.2 to 30.5)					0.1615
NMCST1500, μm	1.8 (−18.5 to 22.2)					0.8581
TMCST500, μm	7.4 (−14.4 to 29.3)					0.4992
TMCST1500, μm	12.6 (−8.0 to 33.2)					0.2257
Cyst volume, μm^3^	−195.8 (− 255.5 to − 136.1)					< 0.0001
SRF, μm	−114.3 (− 159.8 to −68.7)					< 0.0001
	**CRVO (*n*** **= 15)**		**BRVO (*n*** **= 42)**		**Difference between treatment groups**^**c**^	
	**Mean difference (95% CI) from baseline**	***p***^**a**^	**Mean difference (95% CI) from baseline**	***p***^**a**^	**Median difference**^**d**^**(95% CI)**	***P***^**b**^
BCVA	−9.5 (−25.5 to 6.5)	0.2248	6.1 (− 2.8 to 14.9)	0.1739	16.0 (0.0 to 35.0)	0.0798
CRT, μm	− 194.2 (− 340.7 to 47.7)	0.0130	−257.3 (−351.9 to −162.6)	< 0.0001	− 45.5 (− 227.0 to 127.0)	0.6311
CST, μm	15.0 (−30.2 to 60.2)	0.4883	−3.0 (− 25.2 to 19.2)	0.7859	−16.0 (−68.0 to 30.0)	0.4967
NMCST500, μm	25.9 (−10.6 to 62.5)	0.1502	7.9 (−13.3 to 29.0)	0.4556	−11.0 (−54.0 to 30.0)	0.6768
NMCST1500, μm	13.5 (−35.2 to 62.1)	0.5619	−2.3 (− 25.1 to 20.4)	0.8370	−6.5 (− 61.0 to 39.0)	0.7788
TMCST500, μm	7.7 (−56.2 to 71.7)	0.7990	7.3 (−14.1 to 28.7)	0.4943	−11.5 (− 55.0 to 31.0)	0.5259
TMCST1500, μm	19.1 (−38.2 to 76.4)	0.4856	10.3 (−10.8 to 31.3)	0.3302	−11.0 (−60.0 to 48.0)	0.5992
Cyst volume, μm^3^	− 140.1 (−267.5 to −12.7)	0.0335	− 216.2 (− 285.5 to − 146.8)	< 0.0001	−68.0 (− 207.0 to 85.0)	0.3323
SRF, μm	−58.3 (− 116.6 to 0.0)	0.0500	−139.9 (−200.5 to −79.4)	0.0001	−3.0 (− 60.0 to − 52.0)	0.6015
	**Naïve (*n*** **= 31)**		**Previously treated (*n*** **= 26)**		**Difference between treatment groups**^**c**^	
	**Mean difference (95% CI) from baseline**	***p***^**a**^	**Mean difference (95% CI) from baseline**	***p***^**a**^	**Median difference (95% CI)**^**d**^	***P***^**b**^
BCVA	11.0 (0.4 to 21.6)	0.0428	−8.8 (−19.2 to 1.6)	0.0928	−18.0 (−35.0 to −4.0)	0.0129
CRT, μm	− 269.8 (−399.4 to −140.3)	0.0002	−205.9 (−286.9 to − 124.9)	< 0.0001	41.5 (−120.0 to 197.0)	0.6251
CST	1.4 (−23.2 to 25.9)	0.9110	2.2 (−31.1 to 35.5)	0.8932	4.0 (−41.0 to 46.0)	0.8413
NMCST500	12.9 (−8.8 to 34.5)	0.2342	12.3 (−18.8 to 43.5)	0.4218	−3.0 (−40.0 to 33.0)	0.8039
NMCST1500	4.0 (−21.5 to 29.6)	0.7494	−0.8 (−35.4 to 33.8)	0.9620	−9.0 (−52.0 to 32.0)	0.6307
TMCST500	7.6 (−23.0 to 38.3)	0.6137	7.2 (−26.2 to 40.6)	0.6630	0.0 (− 36.0 to 43.0)	0.9872
TMCST1500	21.3 (−5.2 to 47.8)	0.1105	2.2 (−31.7 to 36.1)	0.8951	−4.0 (−47.0 to 39.0)	0.8538
Cyst volume, μm^3^	−218.8 (− 304.5 to − 133.2)	< 0.0001	− 169.2 (−256.9 to −81.5)	0.0005	70.5 (−80.0 to 181.0)	0.3653
SRF, μm	− 128.9 (−202.3 to − 55.5)	0.0015	−92.3 (− 133.9 to − 50.7)	0.0004	0.0 (−64.0 to 48.0)	0.8969

*CRVO* Central retinal vein occlusion, *BRVO* Branch retinal vein occlusion, *CI* Confidence interval, *BCVA* Best corrected visual acuity, *CRT* Central retinal thickness, *CST* Central subfoveal thickness, *NRT* Nasal retinal thickness, *500* ring of 500 μm, *1500* Ring of 1500 μm, *TRT* Temporal retinal thickness, *SRF* Subretinal fluid

^a^Two-way analysis of variance (ANOVA) test

^b^Mann-Whitney U test

^c^First column versus (VS) the second one

^d^Hodges-Lehmann median difference (95% Confidence interval)

Once adjusted by different covariates, there was no significant improvement in BCVA at month-6 according to diagnosis, treatment status, or ELM status (Table 4).

**Table 4:** Adjusted comparison of mean change from baseline to month-6 in different study variables according to baseline diagnosis and according to external limiting membrane (ELM) status. Statistical significance was assessed using the analysis of covariance (ANCOVA)

	**Diagnosis**			*P*
	**CRVO (*n*** **= 15)**	**BRVO (*n*** **= 42)**		
BCVA^a^				
Mean (SEM)	−3.8 (6.3)	4.0 (3.6)		0.2930
95% CI	−16.4 to 8.8	−3.3 to 11.4		
CRT^b^, μm				
Mean (SEM)	−200.2 (74.1)	− 255.1 (43.1)		0.5338
95% CI	− 349.1 to − 51.3	− 341.7 to − 168.5		
CST^c^, mm^3^				
Mean (SEM)	5.5 (19.3)	0.4 (11.2)		0.8218
95% CI	−33.3 to 44.4	− 22.2 to 23.0		
SRF*^d^, μ				
Mean (SEM)	−46.6 (42.3)	− 49.2 (24.2)		0.9583
95% CI	− 133.3 to 40.2	− 98.8 to 0.32		
Cyst volume^e^, μm^3^				
Mean (SEM)	−71.0 (78.7)	−199.3 (44.9)		0.1771
95% CI	− 232.1 to 90.1	− 291.3 to − 107.3		
	**Treatment Status**			
	**Naïve (*n*** **= 31)**	**Previously treated (*n*** **= 27)**		*P*
BCVA^a^				
Mean (SEM)	4.4 (4.5)	−1.0 (4.9)		0.4522
95% CI	−4.6 to 13.4	−10.9 to 9.0		
CRT^b^, μm				
Mean (SEM)	− 308.4 (58.3)	− 165.5 (57.7)		0.0988
95% CI	− 417.5 to −199.3	− 281.3 to − 49.5		
CST^c^, mm^3^				
Mean (SEM)	−2.4 (14.2)	6.3 (15.1)		0.6974
95% CI	−30.8 to 26.1	−23.9 to 36.5		
SRF*^d^, μ				
Mean (SEM)	−22.7 (26.9)	−87.3 (33.2)		0.1498
95% CI	−77.9 to 32.3	−155.3 to 24.7		
Cyst volume^e^, μm^3^				
Mean (SEM)	− 186.0 (50.0)	−136.8 (61.7)		0.5482
95% CI	− 288.3 to −83.6	− 263.2 to − 10.4		
	**ELM status**			
	**Non-disrupted**	**Partially disrupted**	**Disrupted**	***p***
BCVA^a^				
Mean (SEM)	−3.5 (6.8)	4.6 (5.0)	2.3 (5.6)	0.2651
95% CI	−17.2 to 10.1	−5.4 to 14.7	−8.9 to 13.5	
CRT^b^, μm				
Mean (SEM)	− 273.6 (80.6)	− 277.3 (59.3)	− 175.3 (66.1)	0.2783
95% CI	− 435.6 to − 111.7	−396.8 to − 158.1	− 308.2 to − 42.3	
CST^c^, μm				
Mean (SEM)	8.6 (21.0)	−7.3 (15.4)	8.0 (17.2)	0.5796
95% CI	−33.5 to 50.9	−38.3 to 23.8	−26.6 to 42.7	
SRF^*d^, μ				
Mean (SEM)	−19.0 (48.7)	−55.1 (29.2)	−57.7 (41.9)	0.6068
95% CI	− 119.0 to 80.9	−115.1 to 4.9	−143.7 to 28.4	
Cyst volume^e^, μm^3^				
Mean (SEM)	−173.5 (90.6)	− 156.4 (54.4)	−181.6 (78.0)	0.8661
95% CI	− 359.4 to 12.4	−268.0 to −44.8	−341.6 to − 21.6	

Bonferroni correction was applied to the pairwise comparisons

Model 1: “Diagnosis” as factor and age, sex, duration of macular edema, treatment status (naïve or previously treated), type of macular edema (ME), and ELM status as covariates

Model 2: “Treatment status” (naïve or previously treated) as factor and age, sex, duration of macular edema, diagnosis, type of macular edema (ME), and ELM status as covariates

Model 3: “ELM status” as factor and age, sex, duration of RVO-ME, treatment status (naïve or previously treated), type ME, and diagnosis as covariates

*BRVO* Branch retinal vein occlusion, *CRVO* Central retinal vein occlusion, Other: Uveitis and Irvine-gass, *BCVA* Best corrected visual acuity, *SEM* Standard error of the mean, *CI* Confidence interval, *CRT* Central retinal thickness, *MV* Macular volume

^a^Also adjusted by baseline BCVA

^b^Also adjusted by baseline CRT

^c^Also adjusted by baseline CST

^d^Also adjusted by baseline SRF

^e^Also adjusted by baseline volume cysts

^*^In the yes with serous retinal detachment (35 eyes). This variable was not adjusted by type of edema

### Changes in CRT and different anatomic outcomes

**Fig. 1:**
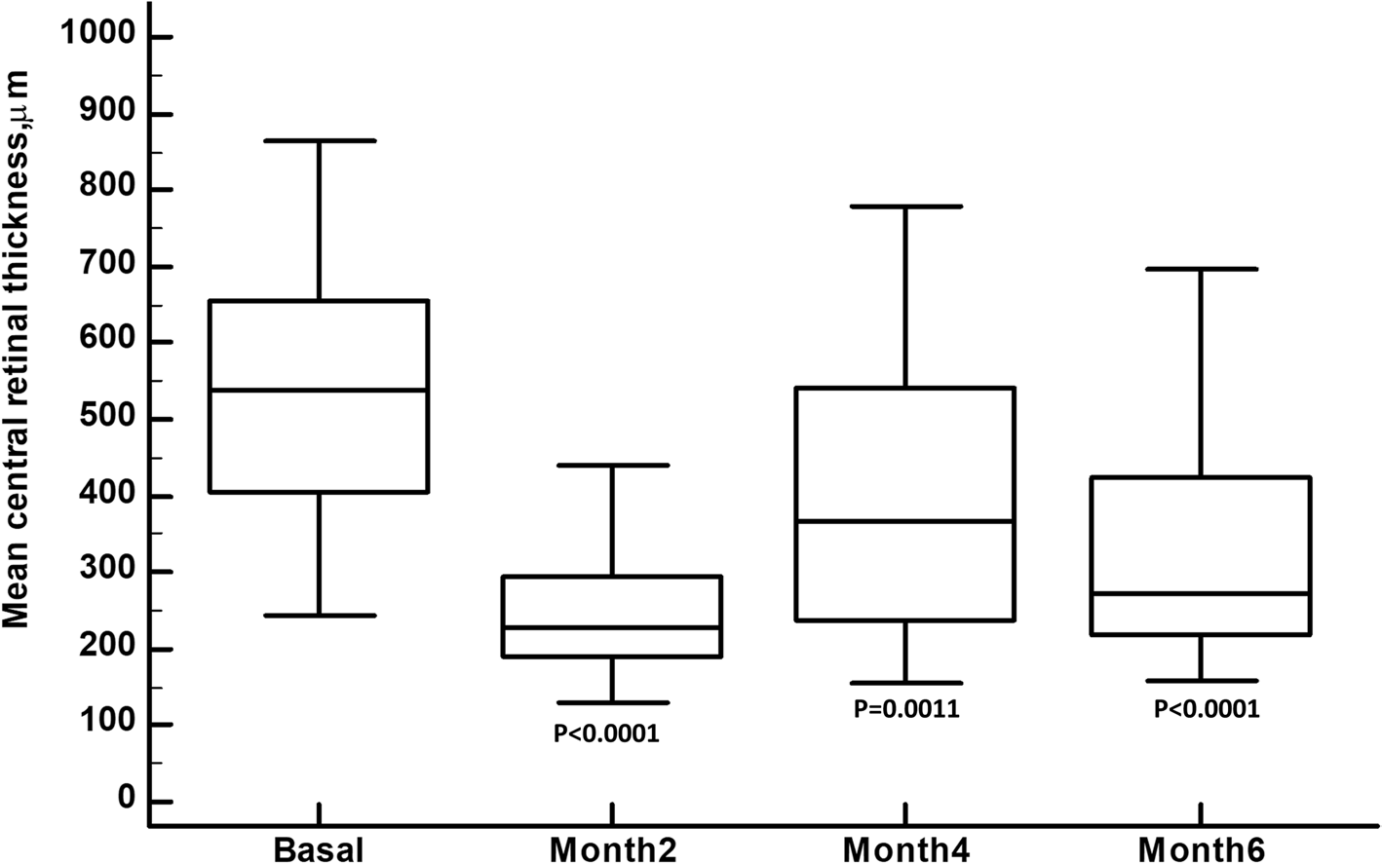
Mean central retinal thickness over the course of the study follow-up. The vertical bars represent the maximun and the minimun values. Intra-group Statistical significance, at the different time point measured, was determined using the Repeated ANOVA test

**Fig. 2:**
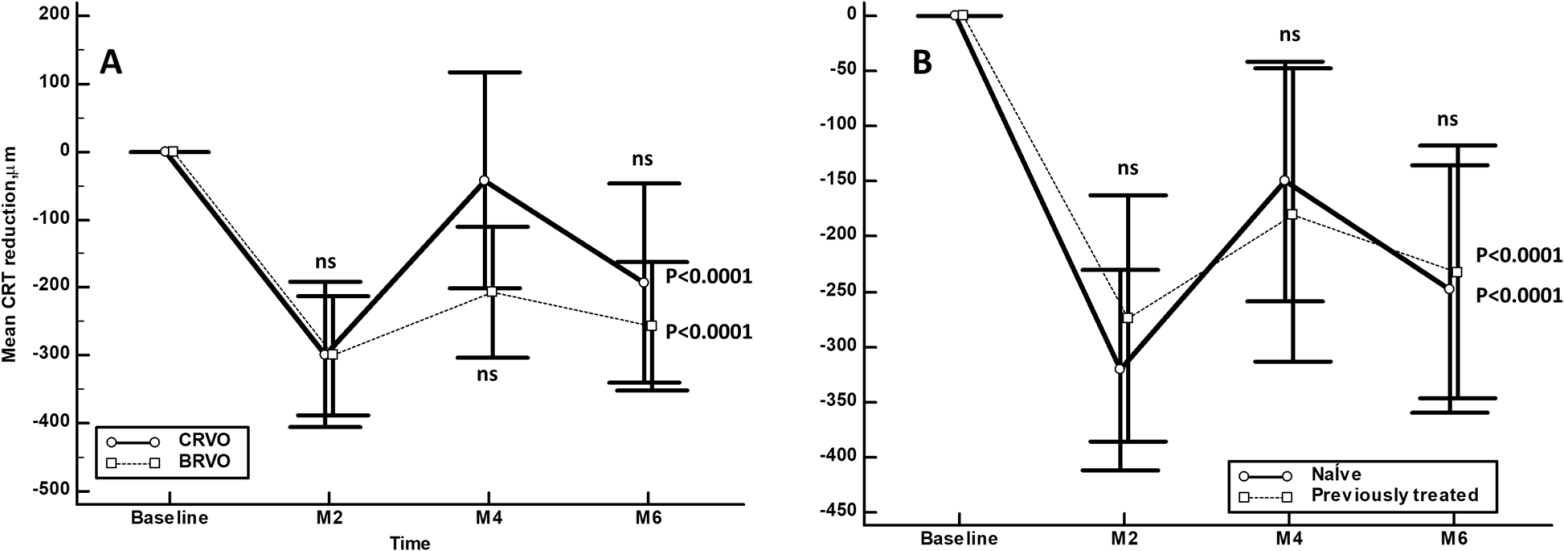
Mean change in central retinal thickness (CRT) from baseline. **A** Comparison between eyes with central retinal vein (CRVO) and those with branch retinal vein (BRVO) occlusion. **B** Comparison between treatment-naïve eyes those who received previous treatment. Statistical significance between groups was determined using the Mann–Whitney U test (statistical significance *P < 0.01). Statistical significance intragroup was determined using the Repeated ANOVA test. CRT: Central retinal thickness; M: Month; CRVO: Central retinal vein occlusion; BRVO: Branch retinal vein occlusion; ns: Not significant

In the overall study sample, CRT was significantly decreased from 567.6 ± 226.2 μm at baseline to 267.7 ± 120.6 μm; 406.4 ± 197.0 μm; and 326.9 ± 141.0 μm at months 2,4, and 6, respectively (*p* < 0.0001, *p* = 0.0011, and *p* < 0.0001, respectively) (Fig. 1). There were no significant differences in CRT reduction, at all the different time points, between CRVO and BRVO eyes (Fig. 2A) or between treatment-naïve eyes and those previously treated (Fig. 2B).

In the overall study population, there was significant reduction in cyst volume, and height of SRF (*p* < 0.0001each, respectively). However, there were no significant changes in nasal, temporal, or subfoveal choroidal thickness (Table 2).

Once adjusted by different covariates, there were no significant differences in CRT or CST mean changes according to diagnosis, treatment status, or ELM status (Table 4).

At month-6, 18 (31.6%) eyes did not show RVO-ME recurrence. Among them, 2 (13.3%) were eyes with CRVO-ME and 16 (38.1%) ones with BRVO-ME, *p* = 0.0787.

The presence of the cysts was significantly reduced in the overall study population, as well as in the eyes with CRVO-ME and BRVO-ME. Additionally, the number of HRF was significantly reduced (*p* = 0.0028) in the CRVO-ME eyes (Table 5). However, ELM status did not change throughout the study (Table 5).

**Table 5:** Overview of optical coherence tomography (OCT) biomarkers over the course of the study. Statistical significance was calculated using the Chi-squared test

	**Overall (*n*** **= 57)**				
	**Baseline**	**Month 2**	**Month 4**	**Month 6**	***p***
DRIL, n (%)					
No	23 (40.4)	25 (43.9)	25 (48.1)	27 (47.4)	0.8365
Yes	34 (59.6)	32 (56.1)	27 (51.9)	30 (52.6)	
HRF, n (%)					
< 10	16 (28.1)	32 (56.1)	25 (48.1)	24 (42.1)	0.0851
10–20	23 (40.4)	13 (22.8)	12 (23.1)	15 (26.3)	
> 20	18 (31.6)	12 (21.1)	15 (28.8)	18 (31.6)	
Cyst, n (%)					
No Cysts	0 (0.0)	31 (54.4)	10 (19.2)	20 (35.1)	< 0.0001
< 100 μm^3^	2 (3.6)	0 (0.0)	0 (0.0)	0 (0.0)	
100–200 μm^3^	12 (21.4)	7 (12.3)	4 (7.7)	4 (7.0)	
> 200 μm^3^	42 (75.0)	19 (33.3)	38 (73.1)	33 (57.9)	
ELM status, n (%)					
Non-disrupted	13 (22.8)	10 (17.9)	8 (15.4)	15 (26.8)	0.8122
Partially disrupted	24 (42.1)	28 (50.0)	25 (48.1)	24 (42.9)	
Disrupted	20 (35.1)	18 (32.1)	19 (36.5)	17 (30.4)	
	**CRVO (*n*** **= 15)**				
	**Baseline**	**Month 2**	**Month 4**	**Month 6**	***p***
DRIL, n (%)					
No	5 (33.3)	2 (13.3)	4 (30.8)	4 (26.7)	0.6078
Yes	10 (66.7)	13 (86.7)	9 (69.2)	11 (73.3)	
HRF, n (%)					
< 10	3 (20.0)	9 (60.0)	5 (38.5)	9 (60.0)	0.0028
10–20	11 (73.3)	4 (26.7)	2 (15.4)	2 (13.3)	
> 20	1 (6.7)	2 (13.3)	6 (46.29	4826.7)	
Cyst, n (%)					
No Cysts	0 (0.0)	9 (60.0)	2 (15.4)	5 (33.3)	0.0016
< 100 μm^3^	1 (6.7)	0 (0.0)	0 (0.0)	0 (0.0)	
100–200 μm^3^	5 (33.3)	0 (0.0)	0 (0.0)	1 (6.7)	
> 200 μm^3^	9 (60.0)	6840.0)	11 (84.6)	9 (60.0)	
ELM status, n (%)					
Non-disrupted	2 (13.3)	3 (21.4)	2 (15.4)	3 (20.0)	0.9950
Partially disrupted	6 (40.0)	5 (35.7)	6 (46.2)	6 (40.0)	
Disrupted	7 (46.7)	6 (42.9)	5 (38.5)	6 (40.0)	
	**BRVO (*n*** **= 42)**				
	**Baseline**	**Month 2**	**Month 4**	**Month 6**	***p***
DRIL, n (%)					
No	18 (42.9)	23 (54.8)	21 (53.8)	23 (54.8)	0.6370
Yes	24 (57.1)	19 (45.2)	18 (46.2)	19 (45.2)	
HRF, n (%)					
< 10	13 (31.0)	23 (54.8)	20 (51.3)	15 (35.7)	0.2755
10–20	12 (28.6)	9 (21.4)	10 (25.6)	13,831.0)	
> 20	17 (40.5)	10 (23.8)	9 (23.1)	14 (33.3)	
Cyst, n (%)					
No Cysts	0 (0.0)	22 (52.4)	8 (20.5)	15 (35.7)	< 0.0001
< 100 μm^3^	1 (2.4)	0 (0.0)	0 (0.0)	0 (0.0)	
100–200 μm^3^	7 (17.1)	7 (16.7)	4 (10.3)	3 (7.1)	
> 200 μm^3^	33 (80.5)	13 (31.0)	27 (69.2)	24 (57.1)	
ELM status, n (%)					
Non-disrupted	11 (26.2)	7 (16.7)	6 (15.4)	12 (29.3)	0.6724
Partially disrupted	18 (42.9)	23 (54.8)	19 (48.7)	18 (43.9)	
Disrupted	13 (31.0)	12 (28.6)	14 (35.9)	11 (26.8)	

*RVO* retinal vein occlusion, *DRIL* Disorganization od inner retinal layers, *HRF* Hyperreflective foci, *CRVO* Central retinal vein occlusion, *BRVO* Branch retinal vein occlusion

**Fig. 3:**
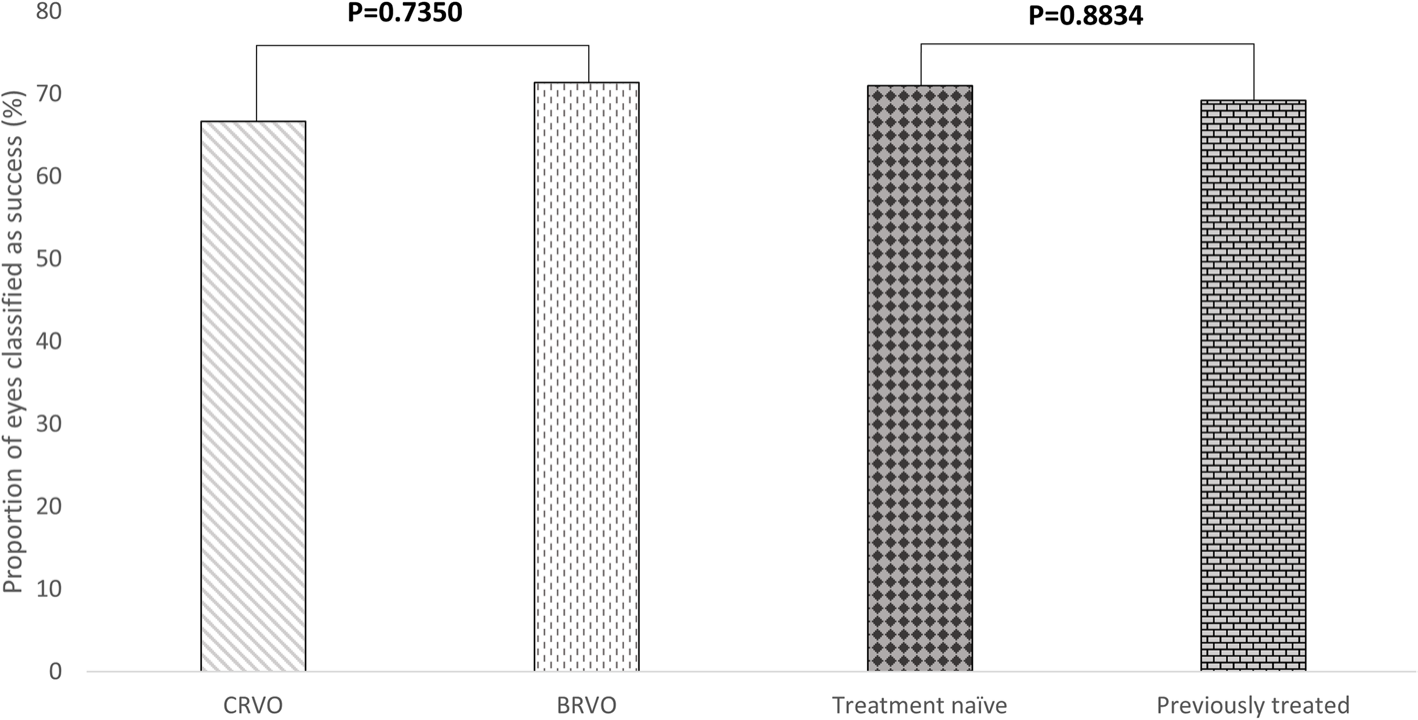
Proportion of eyes classified as success according to diagnosis and treatment status

Forty (70.2%) eyes were classified as anatomic success at month-6, 10 (66.7%) in the eyes with ME secondary to CRVO (CRVO-ME); 30 (71.4%) in those with ME secondary to BRVO (BRVO-ME); 22 (71.0%) in the treatment naïve eyes; and 18 (69.2%) in the previously treated ones (Fig. 3).

There were no significant differences in CTR reduction or in mean change in BCVA between eyes with a RVO-ME duration ≤ median (− 253.4 ± 267.4 μm and 0.9 ± 29.5 letters, respectively) and those with a RVO-ME duration > median (− 227.5 ± 321.8 μm and 3.0 ± 29.0 letters, respectively), *p* = 0.5494 and *p* = 0.8730, respectively (Mann-Whitney U test).

### Factors associated with functional and anatomic outcomes

Logistic regression analysis found that the presence of 10–20 HRF at baseline and the change in septum status (present at baseline versus absent at month-6) were predictors of anatomic success in the univariate analysis; while baseline disrupted ELM and Baseline BCVA were predictors of achieving a BCVA ≥15 letters at month 6. However, in the multivariate analysis only the baseline BCVA was significantly associated with functional success; while none factor was predictor of anatomic success (Table 6).

**Table 6:** Univariate and multivariate analysis of baseline factors associated with anatomic success and best corrected visual acuity improvement ≥15 letters. Factors associated with success in the univariate analysis at *p* ≤ 0.1 were included in the multivariate analysis

Factor	Anatomic success		BCVA gaining ≥ 15 letters	
	Univariate		Univariate	
	OR (95% CI)	***P***	OR (95% CI)	***P***
**Sex**				
**Ref: Women**				
**Men**	0.48 (0.15 to 1.52)	0.2103	2.55 (0.83 to 7.79)	0.1013
**Age**^**a**^				
**> 73 years**	2.2 (0.69 to 7.25)	0.1779	0.57 (0.19 to 1.71	0.3132
**ME duration**^**a**^				
**> 32 days**	0.40 (0.12 to 1.31)	0.1300	1.44 (0.48 to 4.29)	0.5148
**ME Duration**				
**Ref: ≤ 30 days**				
**> 30 ≤ 60**	0.27 (0.05 to 1.39)	0.1177	2.64 (0.57 to 12.25)	0.2555
**> 60**	0.33 (0.09 to 1.22)	0.0955	0.91 (0.26 to 3.139	0.8745
**ME duration**				
**Ref: ≤ 90 days**				
**> 90 days**	0.39 (0.11 to 1.41)	0.1501	0.48 (0.12 to 1.98)	0.3082
**RVO**				
**Ref: CRVO**				
**BRVO**	1.88 (0.54 to 6.50)	0.3193	1.69 (0.46 to 6.23)	0.4287
**ME Subtype**				
**Ref: SLRS**				
**CME**	2.33 (0.12 to 43.79)	0.5711	1.00 (0.98 to 1.02)	0.9981
**SRD**	2.50 (0.14 to 43.97)	0.5311	1.00 (0.98 to 1.02)	0.9981
**Treatment status**				
**Ref: Naïve**				
**Previously treated**	1.29 (0.41 to 4.07)	0.6613	0.36 (0.12 to 1.16)	0.0864
**ELM status**				
**Ref: Non-disrupted**				
**Partially disrupted**	0.73 (0.15 to 3.47)	0.6911	5.69 (0.62 to 52.34)	0.1248
**Disrupted**	0.56 (0.11 to 2.72)	0.4692	19.5 (2.11 to 179.91)	0.0088
**Baseline BCVA**^**a**^				
**> 60**	0.66 (0.21 to 2.06)	0.4702	0.03 (0.00 to 0.21)	0.0007
**DRIL**				
**Ref: No**				
**Yes**	1.36 (0.39 to 4.74)	0.7280	2.84 (0.86 to 9.44)	0.0880
**DRIL Location**				
**Ref: only INL/ONL**				
**Both affected**	1.05 (0.33 to 3.33)	0.9340	0.36 (0.07 to 1.82)	0.2186
**HRF**				
**Ref: < 10**				
**10–20**	4.75 (1.11 to 20.39)	0.0361	0.96 (0.24 to 3.83)	0.9567
**> 20**	2.60 (0.63 to 10.79)	0.1881	1.76 (0.43 to 7.19)	0.4313
**HRF Location**				
**Ref: only INL/ONL**				
**Both affected**	1.01 (0.31 to 3.32)	0.9830	0.72 (0.23 to 2.23)	0.5684
**SRF**				
**Per μm thicker**	1.00 (0.99 to 1.01)	0.4303	0.99 (0.99 to 1.01)	0.6753
**SRF**				
**> 119** μm	2.97 (0.62 to 14.22)	0.1732	3.11(0.72 to 13.44)	0.1285
**Cysts**				
**Ref: < 100**				
**100–200**	1.00 (0.05 to 19.96)	1.000	1.00 (0.98 to 1.02)	0.9981
**> 200**	3.67 (0.21 to 64.55)	0.3746	0.99 (0.99 to 1.01)	0.9979
**Change in Septum M6**				
**Ref: No**				
**Yes**	13.93 (2.78 to 69.83)	0.0014	1.97 (0.65 to 5.959	0.2300

Multivariate analysis:

• Anatomic success:

○ MD Duration > 60 days: OR: 0.34; 95% CI: 0.08 to 1.35; *p* = 0.1234

○ HRF 10–20: OR: 4.26; 95% CI: 0.88 to 25.60; *p* = 0.0712

○ Change in Septum month 6: OR: 1.09; 95% CI: 0.31 to 3.83; *p* = 0.8939

• BCVA gaining ≥15 letters:

○ Baseline BCVA: OR: 0.04; 95% CI: 0.00 to 0.39; *p* = 0.0057

○ Treatment status: OR: 0.46; 95% CI: 0.19 to 1.96; *p* = 0.3003

○ ELM status (Disrupted): 5.78; 95% CI: 0.48 to 69.77; *p* = 0.1670

○ DRIL: OR: 1.29; 95% CI: 0.26 to 6.54; *p* = 0.7571

Age: OR: 2.96; 95% CI: 0.92 to 9.57; *p* = 0.0695

Duration of ME: OR: 2.65; 95% CI: 0.82 to 8.58; *p* = 0.1036

Abbreviations: *OR* Odds ratio, *CI* Confidence interval, *ME* Macular edema, *SLRS* Sponge-like retinal swelling, *CME* Cystoid macular edema, *SRD* Serous retinal detachment, *DRIL.* Disorganization of inner retinal layer, *INL* Inner nuclear layer, *ONL* Outer nuclear layer, *HRF* Hyperreflective foci, *SRF* Subretinal fluid, *M* Month

^a^Reference group ≤ Median

The mean number of DEX implant administered throughout the study was 1.46 ± 0.50. Twenty-one (36.8%) eyes underwent only one DEX implant during the study follow-up, while 36 (63.2%) required rescue therapy (27 eyes received an additional DEX implant and 9 eyes received anti-VEGF injections), with a median time interval of 109 (95% CI: 72 to 148) days.

### Safety

No patient experienced a significant increase (≥ 5 mmHg) in IOP.

## Discussion

The results of the current study showed that the DEX implant Ozurdex® significantly reduce the CRT in patients with RVO-ME, with no differences between eyes with BRVO-ME and those with CRVO-ME or between treatment naïve and previously treated eyes.

Regarding function, there was a BCVA mean gain of + 5.4 letters at month-2, and at month-6, 20 (35.1%) eyes had achieved a BCVA improvement ≥15 letters. Although there was no difference in mean change in BCVA between eyes with CRVO-ME and those with BRVO-ME, the mean change in BCVA was significantly greater in the treatment naïve eyes than in the previously treated ones.

Additionally, 40 (70.2%) eyes were classified as anatomic success at month-6 (CRT ≤ 250 μm or CRT reduction > 10%, at the end of the follow-up period), with no differences between CRVO/BRVO (mean difference 4.7%, *p* = 0.7350) and between naïve/previously treated (men difference 1.8%, *p* = 0.8834) groups.

DEX implant was the first medical therapy approved for treatment of RVO-ME. Different studies have shown clinically significant functional and anatomic improvements after the administration of the DEX implant in patients with RVO [7, 8, 21–25].

However, these studies did not provide information about potential biomarkers that can predict clinical outcomes in RVO-ME patients.

In a previous study published by our group we found a significant improvement of ELM integrity after DEX implant administration in patients with ME secondary to retinal vascular disease (diabetic macular edema or RVO-ME) [14]. However, this study failed to confirm that finding. It may be partially explained by the fact that our previous study included eyes with diabetic macular edema, while the current study only included eyes with RVO. Additionally, in our previous study [14] we assessed the changes in ELM from a quantitative point of view, while in the current one the changes in ELM have been qualitatively evaluated.

In the current study, we found significant differences in baseline BCVA depending on ELM status. This may be due to the fact that even though there was an anatomical improvement, there is a tipping point from which photoreceptor damage cannot be recovered, which would highlight the relevance of an early therapeutic approach.

Regarding the effect of DEX implant on cysts, as compare to baseline (0/57 eyes), we found a greater proportion of eyes without cysts at month 2 (31/57, *p* < 0.0001), month 4 (10/57, *p* = 0.0005), and month 6 (20/57, *p* < 0.0001). However, no significant changes were observed in DRIL and/or HRF.

It is no easy to compare our studies with the currently available scientific evidence, since most of the evidence evaluated ME secondary to diabetic retinopathy (DME).

Despite the association between OCT biomarkers and BCVA at baseline, the current study did not observe any relationship between the different OCT biomarkers (DRIL, HRF, cyst, and ELM status) evaluated at baseline and the clinical outcomes.

We found worse baseline BCVA in those eyes with thicker SRF, presence of DRIL, disrupted ELM, or HRF > 20. However, due probably to the limited sample, we did not find significant associations between these OCT biomarkers and the changes in BCVA.

Evidence regarding the importance of HRF is eluding. In our study a significant association between HRF and final BCVA has not been established; however, we found worse baseline BCVA in eyes with over 20 HRF, and also in eyes with more disrupted ELM. Although we do not localize the HRF within the retinal layers, it is known that a disrupted ELM cannot block the migration of extravasated lipoproteins in the inner retinal layers to the outer retinal layers of the ELM, and makes it possible to pass these extravasated blood constituents of the HRF through the outer retinal layer [26].

The relationship between SRF and visual function is controversial.

Larger intraretinal cysts, greater amount of SRF, higher percentage of DRIL, and percentage of ELM disruption have been associated with worse baseline BCVA in patients with RVO-ME. However, only percentage of ELM disruption independently impacted baseline BCVA [27].

The results of the current study are in agreement with a previous study conducted by our group that found no differences in BCVA improvements between the eyes with severe ELM disruptions at baseline and those with no or less severe ELM disruption at baseline [14].

Since we found significant associations between different OCT biomarkers (DRIL, ELM status, height of SRF, or HRF) and the baseline BCVA, the lack of relationship between these biomarkers and clinical outcomes needs to be further investigated.

Moreover, patients with good initial VA were more likely to achieve a BCVA improvement ≥15 letters. This finding demonstrates superior visual outcomes in those eyes with good initial vision, suggesting that initiating treatment as soon as BCVA begins to decline is of utmost importance in the treatment of RVO-ME.

CRT was significantly reduced at all the different time-point measures, not only in the overall study populations, but also in the CRVO and BRVO eyes, and in the treatment naïve and previously treated ones. We did not find any differences at any of the different time-points according to the diagnosis (CRVO vs BRVO) or to the treatment status (treatment naïve vs previously treated).

This study did not find any relationship between the time elapsed from RVO diagnosis to the first intravitreal DEX implant and the changes in CRT or BCVA. Conversely, the LOUVRE study found that the mean gain in BCVA was greater in patients with recent-onset ME [28]. Such a difference may be explained by the fact that in our study the median time from diagnosis to treatment was 32 days, while in the LOUVRE study the cut-off point was established in 3 months. In our study 13 (22.8%) eyes had duration of ME at baseline ≥90 days. Although CRT reduction and BCVA improvement were lower in those eyes with ME duration ≥90 days (163.5 ± 261.6 μm and 7.0 ± 27.6 letters, respectively) than in those with ME duration < 90 days (263.5 ± 300.6 μm and 4.6 ± 29.2 letters, respectively), such differences were no statistically significant (*p* = 0.3816 and *p* = 0.1558, respectively, Mann-Whitney U test).

Although in patients with RVO-ME the DEX implant has been associated with an elevation of IOP [7, 8, 28], in the current study none eye has experienced an IOP increased ≥5 mmHg.

Several limitations should be taken into consideration when interpreting the results of this study. The main one is its retrospective design, which entail potential bias and confounding factors. In order to minimize its impact, we applied strict inclusion/exclusion criteria. The second limitation is the lack of sample size calculation. In fact, the power for detecting the observed differences in BCVA change, between baseline and month-6, was 13% in CRVO vs BRVO eyes and 97% between treatment naïve and previously treated eyes. Additionally, the power for detecting the observed differences in CRT reduction at month 6, between CRVO vs BRVO and between treatment naïve vs previously treated eyes, was 13% each, respectively.

Although of the total amount of follow-up measurements there were only 4% of missing values and that they were allocated using an algorithm of multiple imputation, it should be taken into consideration when interpreting the results.

An additional limitation is the fact that we have measured the presence or absence of DRIL, but not whether their extension changed over the course of the study. This fact, therefore, may explain the lack of significant changes after treatment. Finally, we did not evaluate the safety profile of DEX, but treatment related adverse events associated with DEX are well known and have been exhaustively reported [29].

## Conclusions

Despite these limitations, the results of this study suggest a positive impact of DEX on CRT in eyes with RVO-ME. DEX implant significantly reduced the presence of cysts and their volume. Additionally, greater amount of SRF, presence of DRIL, presence of HRF (> 20), or disrupted ELM were significantly associated with worse baseline BCVA.

We also found a significant improvement in BCVA at month-2 after DEX implantation and approximately 1/3 of the eyes showed no recurrence of the ME. We did not find any relationship between the OCT biomarkers and the clinical outcomes, although those eyes with good initial VA were more likely to achieve a BCVA improvement ≥ letters.

In order to identify the best patient profile candidate to benefit from DEX treatment, further research will be needed to elucidate the complex relationship between the different OCT biomarkers and the clinical outcomes.

## Data Availability

The data that support the findings of this study are available from the corresponding author, [VCN], upon reasonable request.

## Abbreviations

ANCOVA: Analysis of covariance
ANOVA: Analysis of variance
Anti-VEGF: Vascular endothelial growth factors inhibitors
BRVO: Branch retinal vein occlusion
BCVA: Best corrected visual acuity
CATT: Comparison of Age-related Macular Degeneration Treatments Trials
CI: Confidence interval
CME: Cystoid macular edema
CRT: Central retinal thickness
CRVO: Central retinal vein occlusion
CST: Subfoveal choroidal thickness
CTN: Nasal choroidal Thickness
CTT: Temporal choroidal thickness
DEX: Dexamethasone intravitreal implant
DRIL: Disorganization of the retinal inner layers
ELM: External limiting membrane
ETDRS: Early Treatment Diabetic Retinopathy Study
EZ: Ellipsoid zone
HRF: Hyperreflective foci
INL: Internal limiting membrane
IRF: Intraretinal fluid
ME: Macular edema
OCT: Optical coherence tomography
RVO: Retinal vein occlusion
RVO-ME: Macular edema secondary to retinal vein occlusion
SD: Standard Deviation
SDm: Spectral domain
SE: Standard error
SLRS: Sponge-like retinal swelling
SRD: Serous retinal detachment
SRF: Subretinal fluid
VA: Visual acuity
VEGF: Vascular endothelial growth factor
